# SjTat-TPI facilitates adaptive T-cell responses and reduces hepatic pathology during *Schistosoma japonicum* infection in BALB/c mice

**DOI:** 10.1186/s13071-015-1275-6

**Published:** 2015-12-30

**Authors:** Wenyue Zhang, Xiaofeng Luo, Fan Zhang, Yuxiao Zhu, Bingya Yang, Min Hou, Zhipeng Xu, Chuanxin Yu, Yingying Chen, Lin Chen, Minjun Ji

**Affiliations:** Department of Pathogen Biology, Nanjing Medical University, Nanjing, Jiangsu 210029 China; Jiangsu Province Key Laboratory of Modern Pathogen Biology, Nanjing, Jiangsu 210029 China; Jiangsu Institute of Parasitic Diseases, Wuxi, Jiangsu 214064 China

**Keywords:** *Schistosoma japonicum*, Vaccine, Th1, Tc1, Triosephosphate isomeras, HIV Tat

## Abstract

**Background:**

Schistosomiasis is a kind of parasitic zoonoses which causes serious damage to public health and social development. China is one of the countries most affected by *Schistosoma japonicum* and an effective vaccine is still needed. In this study, we adopted Tat-mediated protein transduction technology to investigate the impact of different antigen presented approaches on host’s immune response and the potential protection against *Schistosoma japonicum* infection.

**Results:**

We successfully constructed the recombinant *S. japonicum* triosephosphate isomerase, Tat-TPI, as a vaccine candidate. Whether injected with Tat-TPI in foot pad or vaccinated with Tat-TPI in the back subcutaneously for three times, the draining popliteal lymph nodes and spleen both developed a stronger CD8^+^T response (Tc1) in mice. Not only that, but it also helped CD4^+^T cells to produce more IFN-γ than TPI immunisation. In addition, it could boost IgG production, especially IgG1 subclass. Most importantly, Tat-TPI immunisation led to the significant smaller area of a single egg granuloma in the livers as compared with TPI-vaccinated or control groups. However, the anti-infection efficiency induced by Tat-TPI was still restricted.

**Conclusion:**

This study indicated that immunisation with Tat-fused TPI could contribute to enhance CD4^+^T-cell response and decrease hepatic egg granulomatous area after *S. japonicum* infection though it did not achieve our expected protection against *Schistosoma japonicum* infection. The optimal vaccine strategy warrants further research.

## Background

Schistosomiasis is still a significant public health problem in tropical and subtropical regions [1]. Although in the past six decades, a series of comprehensive measures have been adopted to control schistosomiasis japonica in China, there remain many challenges we need to face. According to conservative estimates, about 0.29 million people are infected and more than 240 million people living in 180 counties remain endemic for *S. japonicum* in China today [2]. Transmitted by *Oncomelania hupensis*, *S. japonicum* could cause serious pathology damage to the liver and intestine. Praziquantel is the effective drug for treatment, but it does not prevent post-treatment reinfection and long-term application might lead to increase the drug resistance [3, 4]. Some other measures including environmental and health-care management and snail control have achieved great success in controlling, but the factors of reinfection, such as many reservoir hosts, still exist [5]. Therefore, development of safe and effective vaccines, especially for veterinary use, is a high priority.

The radiation-attenuated (RA) vaccine can induce high protection against schistosome infection in many experimental animal models [6, 7]. However, the requirement of large amounts of cercariae, potential side effects and the risk of reinfection limited its application in the population. Our previous experiments showed that three vaccinations with RA cercariae could induce high levels of worm reduction rate (77.62 %) and hepatic egg reduction rate (88.8 %) in pigs [7]. In this case, antibody and CD4^+^T-cell-mediated, IFN-γ-dependent effector mechanisms are the important reason for high protection [8–10]. We also found that about one week after immunisation with RA cercariae, some gene expression of killer factors like granzymes and IFN-γ was enhanced in the skin draining lymph nodes. This implies that CD8^+^T-cells might be related to radiation-induced high protection. Similarly, Pancré et al. found that CTL response caused by CD8^+^T-cells could participate in the protection mechanism against *S. mansoni* infection [11, 12]. Thus, we expect that the cooperation between CD8^+^T cells, CD4^+^T cells and IgG responses would achieve an effective protection against schistosomiasis.

Triosephosphate isomerase (TPI) is one of the World Health Organisation (WHO) recommended schistosome vaccine candidate molecules. It is a kind of glycolytic enzyme, involved in the process of glucose metabolism [13]. TPI is distributed in all developmental stages of schistosome life-cycle. It was reported that SjTPI immunised mice together with Freund's adjuvant induce a 57.8 % liver egg reduction rate [14], while DNA vaccines containing the optimised SjTPI gene (SjTPI.opt) was able to induce 36–39 % reductions in worm burden [13, 15]. Protein transduction domain (PTDs), also known as cell-permeable peptides (CPPs), refers to a class of small (<20 amino acids), relatively nontoxic peptides, which are capable of not only crossing the cell membrane themselves but also carrying many various small molecules into cells, including siRNA, plasmid DNA, combinant proteins, viruses and other different nanoparticles [16]. Tat, the HIV transactivator of transcription protein, is the most widely used PTDs. Tat-fused protein could efficiently transduce dendritic cells (DCs) and was processed by proteasomes for MHC class I-dependent presentation to CD8^+^T lymphocytes [17]. In this study, we evaluated the immunogenicity and efficacy of the fusion protein containing the protein transduction domain of HIV-1 TAT and the *Schistosoma japonicum* antigen Sj-TPI in a mouse model. The approach we used was to express this fused antigen to facilitate the entry of protein into cells and enhance CD4^+^ and CD8^+^ T-cell response.

## Methods

### Ethics statement

The animal experiments were approved by the Nanjing Medical University Animal Ethics Committee. All the mice were subjected to minimum suffering.

### Animals and parasites

Female BALB/c mice, 6–8 week-old, were obtained from Comparative Medicine Center of Yangzhou University (Yangzhou, China) and kept in specific pathogen-free environment for experimental use. Snails infected with *Schistosoma japonicum* (a Chinese strain of *S. japonicum*-infected *Oncomelania hupensis*) were purchased from the Jiangsu Institute of Parasitic Diseases (Wuxi, China). Cercariae were collected from infected snails.

### Prokaryotic expression vector construction and expression of the fusion protein Tat-TPI and TPI

The plasmid, pcDNA3.1-SjTPI.opt [15, 18], containing the optimised triosephosphate isomerase gene of *S. japonicum*, was provided by the Jiangsu Institute of Parasitic Diseases (Wuxi, China). Then according to the nucleic acid sequences of Tat-TPI and TPI, the following primers were designed: (i) Tat-TPI: forward primer: 5’-CGC GGA TCC TAT GGC AGG AAG AAG CGG AGA CAG CGA CGA AGA AGC AGC AGC CGG AAG TT-3’; (ii) TPI, forward primer: 5’-CGC GGA TCC AGC AGC AGC CGG AAG TT-3’; (iii) reverse primers for Tat-TPI and TPI: 5’-CCC AAG CTT TCA CTG CCG GGC CTT G-3’. The gene fragments of Tat-TPI and TPI were amplified by PCR. These two genes were respectively cloned into the His-tagged protein downstream of expression vector pET-32a(+) and the recombinant plasmids pET-32a(+)-Tat-TPI and pET-32a(+)-TPI were obtained. The recombinant plasmids were transformed into *E. coli* strain BMRosetta (DE3) for protein expression. After induced expression by IPTG for 4 h at 25 °C and subsequent purification with Ni-NTA Sepharose FF, the purified fusion protein Tat-TPI and TPI were obtained.

### Immune reaction of the draining lymph nodes

To ascertain the immune response induced by TAT-coupled protein in mice, mouse foot pads of each group were injected subcutaneously with 50 μg Tat-TPI, TPI in 20 μl PBS per side, respectively. Negative controls were injected with isometric PBS. The draining popliteal lymph nodes were aseptically removed 4 days after vaccination, then lymphocytes were prepared for flow cytometry detection. The experiments were duplicated twice.

### Antibody-blocking assay

In order to understand the effect of CD8^+^T cells on CD4^+^T-cell response, we performed antibody blocking experiments in vitro. The single spleen cell suspensions were incubated in 24-well flat-bottom plates (1 ml/well, 2 × 10^6^ cells/ml). For CD8^+^T cells blocking experiments, spleen T cells were treated with 5 mg/ml of anti-mouse CD8a antibody (BioLegend, 100735, Canada) or isotype control antibody IgG2a for 1 h at 37 °C. Then Tat-TPI, TPI (100 mg/ml) and PBS were added, respectively, and incubated at 37 °C, 5 % CO_2_. After 18 h incubation, T cells were restimulated for 6 h at 37 °C in 5 % CO_2_ with 2 μl Leukocyte Activation Cocktail (BD Biosciences). Finally, cells were collected for flow cytometry detection of Th1 expression. The experiments were repeated four times.

### Animal immunisation and challenge experiment

Mice were randomly divided into four groups (20 mice/group). The antigens Tat-TPI and TPI were administered subcutaneously (100 μg/mice) emulsified in the same volume of incomplete Freund’s adjuvant (IFA) per mouse in corresponding groups on days 1, 14 and 28. The other two groups were injected isopyknic IFA and PBS with the same immune process. Two weeks after the third immunisation, eight mice in each group were sacrificed for flow cytometry assay and serum samples were collected for antibody analysis. The remaining mice were percutaneously challenged with 40 ± 1 *S. japonicum* cercariae. All animals were sacrificed at 6 weeks post-infection and parasite burden, T-cell responses were observed. Parasite burden was evaluated by adult worm recovery, egg burdens and area of single egg granuloma in the livers. Briefly, the adult worms were collected and counted after perfusion of the portal vein with PBS. Meanwhile, the liver was isolated, weighed and digested with 10 ml 5 % KOH at 37 °C for 24 h and eggs counted in 10 μl liver digest solution. Each liver sample was counted 3 times and the mean count was used as eggs per gram (EPG) in mice. The sectioned liver tissue (1–5 cm^3^) were fixed in 4 % paraformaldehyde, embedded in paraffin and stained with haematoxilin and eosin, according to standard protocols. Single-egg granulomas were examined and their sizes were calculated using AxioVision Rel 4.7 (Carl Zeiss GmbH, Jena, Germany). At least fifteen single egg granulomas per liver section were photographed. Additionally, the percentage of Th1 and Tc1 in the spleens was detected by flow cytometry.

### Measurement of specific antibodies response

ELISA was applied to detect schistosome-specific antibody responses. Briefly, 96-well plates (Costar) were coated with TPI (10 μg/ml) at 4 °C overnight, then washed 5 times with PBS-0.05 % Tween-20 (PBST) and blocked in PBST-5 % skimmed milk at 37 °C for 2 h. After washing 5 times again, each well was incubated with 100 μl sera samples diluted 1:100 with PBS for 2 h at 37 °C. Next, plates were washed with PBST and incubated with 100 μl HRP-conjugated goat-anti-mouse IgG, IgG1 and IgG2a (diluted 1:5000, Biosharp) for 1 h at 37 °C. Lastly, after five washes with PBST, each well plate was developed with 100 μl tetramethylbenzidine (TMB, 10 mg/ml) for 20 min at 37 °C and the reaction was stopped by adding 50 μl H_2_SO_4_ (2 M). The optical density (OD) was read at 450 nm using an ELISA reader (Bio-TEK, USA).

### Flow cytometry detection

Flow cytometry was used to analyse the percentages of Th1, Th2, Tc1, Tc2 in the draining popliteal lymph nodes or spleens after immunisation or infection *ex vivo* and in vitro CD8^+^T cell-blocking experiment. Single cell suspensions were prepared by collecting the cells after anti-CD8 antibody blocking, or grinding popliteal lymph nodes or spleens in incomplete RPMI 1 × 1640 medium (WISENT, Canada) followed by red blood cell (RBC) lysis, then 2 × 10^6^ cells per ml were stimulated for 6 h at 37 °C in 5 % CO_2_ with 2 μl Leukocyte Activation Cocktail with BD GolgiPlug (BD Biosciences, USA) in 24-well plates (1 ml/well). Next, 1 × 10^6^ cells were stained in 100 μl of Staining Buffer [PBS containing 1 % FBS (Gibco, USA)] with following fluorochrome-conjugated monoclonal antibodies specific for surface antigen: CD3e-APC (BD Biosciences), CD4-FITC (BD Biosciences), CD8a-FITC (BD Biosciences), APC-labelled Hamster IgG1 (BD Biosciences) and FITC-labeled Rat IgG2a isotype control antibodies (BD Biosciences) for 30 min at 4 °C in the dark. The cells were washed twice with Staining Buffer, then fixed and permeabilised with Cytofix/Cytoperm Fixation/Permeabilisation Solution Kit (BD Biosciences) following the manufacturer’s instructions. Subsequently, cells were incubated for 30 min at 4 °C in the dark with the following monoclonal antibodies: IFN-γ-PE (BD Biosciences), IL-4-PE (BD Biosciences) and PE-labeled Rat IgG1 isotype control antibody (BD Biosciences). Finally, cells were washed twice with 1 × Perm/Wash buffer (BD Biosciences) and resuspended in Staining Buffer prior to flow cytometry analysis.

### Statistics

Differences between groups were analysed by one-way analysis of variance (ANOVA) using GraphPad Prism version 5.01 for Windows. Significance was considered when *P* values ≤ 0.05 or 0.01.

## Results

### Production and characterisation of recombinant Tat-TPI and TPI proteins

**Fig. 1:**
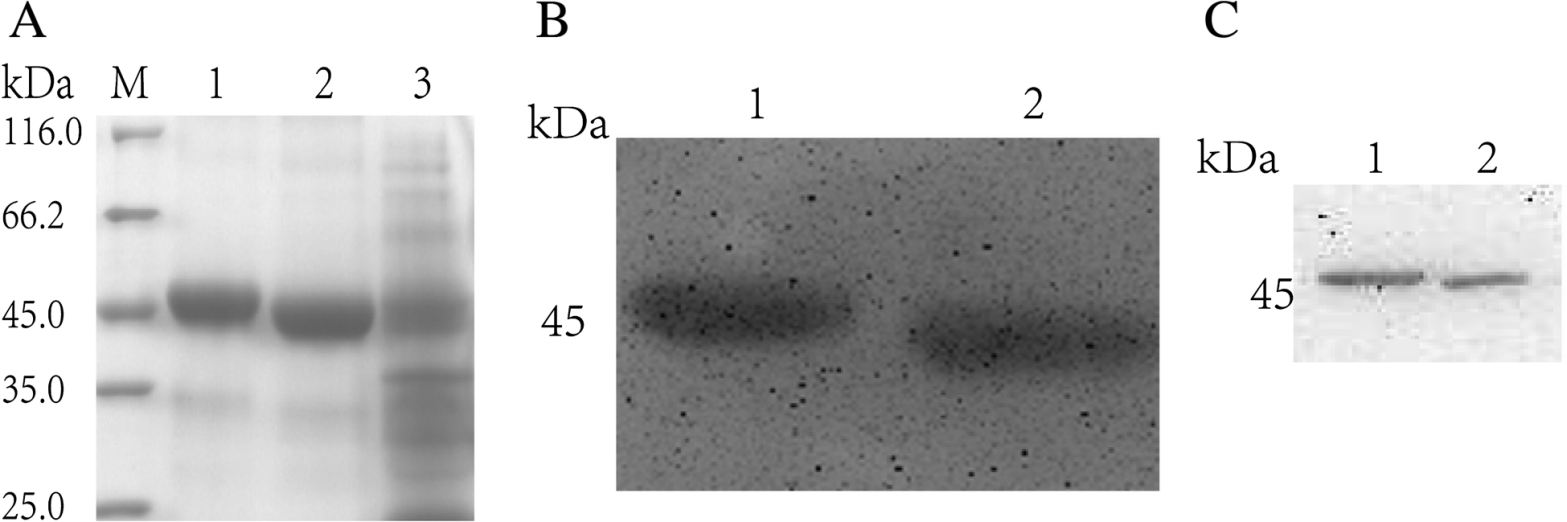
Expression, purification and identification of the fusion proteins Tat-TPI and TPI. **a**. Purification of two fusion proteins detected by Protein Gel Electrophoresis. M: molecular weight marker, Lane 1: recombinant SjTat-TPI, Lane 2: recombinant SjTPI, Lane 3: the recombinant plasmid without purification. **b**. Fusion proteins recognised by His-Ab with Western blotting. Lane 1: recombinant SjTat-TPI, Lane 2: recombinant SjTPI. **c**. Fusion proteins recognised by *S. japonicum* infected-mice serum with Western blotting. Lane 1: recombinant SjTat-TPI, Lane 2: recombinant SjTPI

After PCR amplification, we obtained the gene fragment of T-TPI (about 807 bp long), slightly longer than the TPI fragment. The recombinant plasmids were constructed successfully, and the recombinant proteins were purified to obtain fusion protein. By SDS-PAGE electrophoresis, the protein bands appeared at about 46 kDa. The fusion proteins of T-TPI and TPI were also recognised by His-Ab and *S. japonicum* infected-mice serum respectively in Western blotting (Fig. 1).

### Immune response in the draining popliteal lymph nodes

**Fig. 2:**
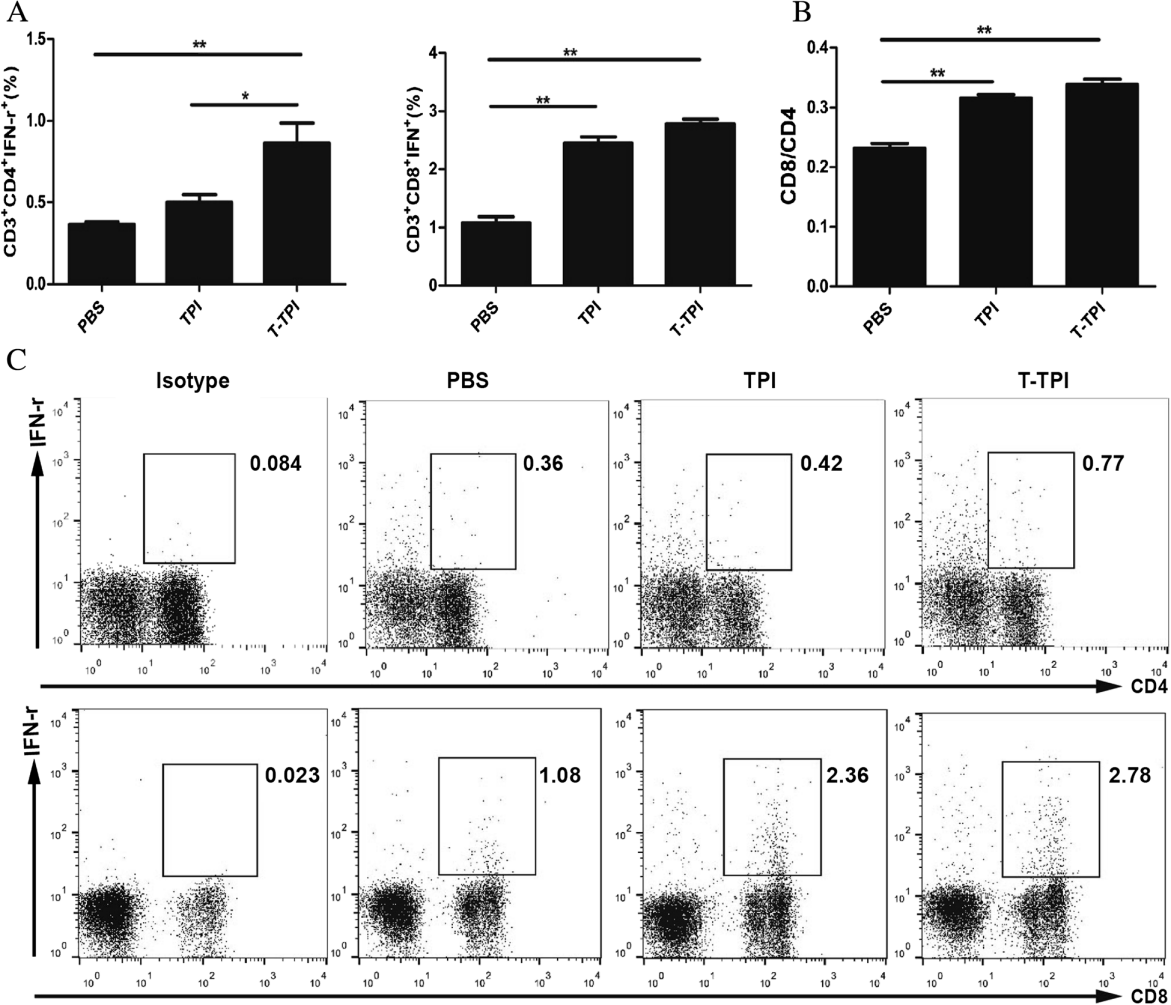
Immune responses in the draining popliteal lymph nodes of mice induced by Tat-TPI (T-TPI) and TPI proteins. **a** and **c**. Percentages of CD4^+^IFN-γ^+^ cells (Th1), CD8^+^IFN-γ^+^ cells (Tc1) analysed by FACS. **b**. The ratio of CD4^+^ T cells to CD8^+^ T cells (CD4/CD8) in the draining popliteal lymph nodes. Data are presented as the means ± SEM from six mice in each group. (*P < 0.05; **P < 0.01)

To investigate the immune effect induced by Tat-TPI and TPI, we injected these two proteins to mice footpad respectively, and observed T-cell response in the draining popliteal lymph nodes. On day 4 post injection, Tat-TPI and TPI vaccination can both evoke host’s immune response. The results showed that Tat-TPI and TPI immunisation in foot pad of mice could induce higher levels Tc1 response in the draining popliteal lymph nodes compared to PBS controls (*P* < 0.01). More importantly, CD4^+^IFN-γ^+^ T-cell response could be quickly evoked by Tat-TPI instead of TPI. There was a significant difference in Tat-TPI- and TPI-vaccinated groups on CD4^+^IFN-γ^+^ T-cell percentages in the popliteal lymph nodes (*P* < 0.05) but not on CD8^+^IFN-γ^+^ T-cell percentages (Fig. 2a, and c).

### CD8^+^ T cells-blocking experiment

**Fig. 3:**
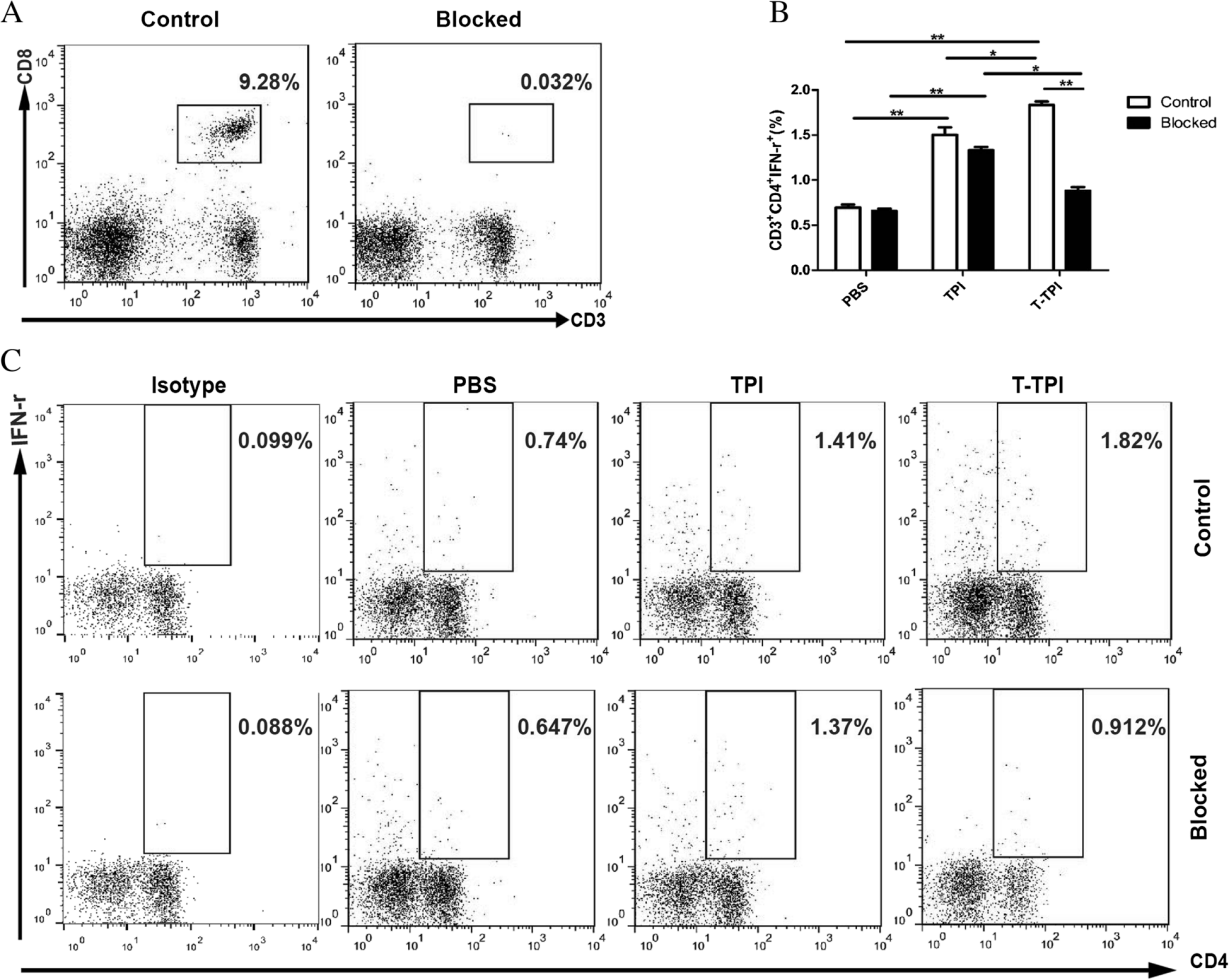
Th1 immune response after CD8^+^T cells blockage in vitro. **a**. CD3^+^CD8^+^T cells were successfully blocked. The blocking efficiency was more than 99.6 %. **b**, **c**. Percentages of CD4^+^IFN-γ^+^ (Th1) in Tat-TPI (T-TPI), TPI stimulated splenocytes with or without CD8^+^T-cell blockage. Data are presented as the means ± SEM from four independent experiments. (*P < 0.05; **P < 0.01)

To ascertain if CD8^+^ T cells could help Th1 response, we used anti-mouse CD8a antibody to block CD8^+^ T cells. Flow cytometry showed Tat-TPI stimulated splenocytes produced higher CD3^+^CD4^+^IFN-γ^+^ cells than TPI stimulation. When CD8^+^ T cells were blocked (Fig. 3a), there was no significant influence on the percentages of CD4^+^IFN-γ^+^ T cells in splenocytes with TPI and PBS stimulation; however, T-TPI stimulated spleen cells expressed lower levels of CD4^+^ IFN-γ^+^ cells than those without CD8^+^ T-cell blockage (*P* < 0.05) (Fig. 3b, and c).

### A high T-cell and antibody response induced by Tat-TPI before challenge

**Fig. 4:**
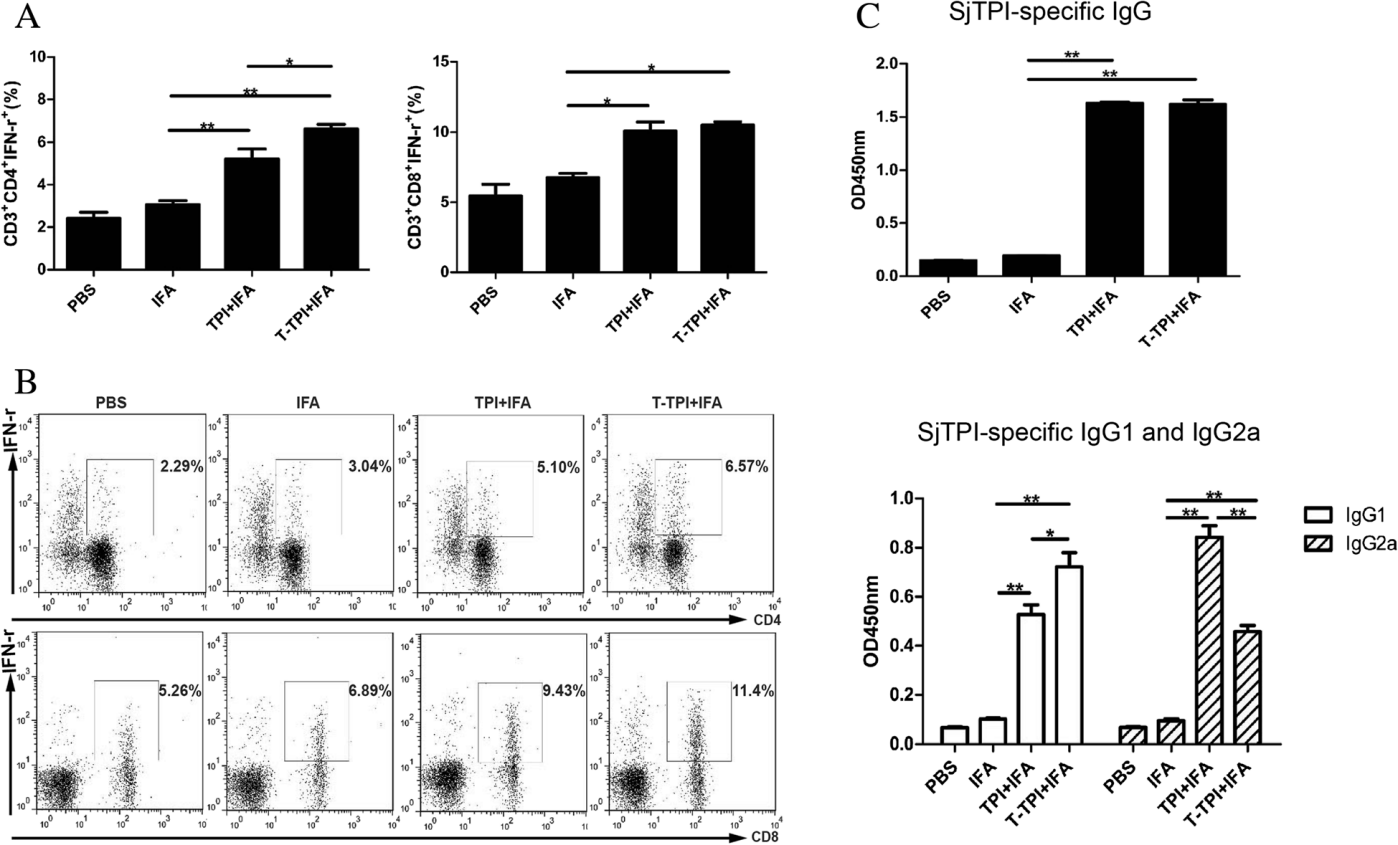
T cell and antibodies responses after three immunisations with T-TPI + IFA, TPI + IFA, IFA and PBS. **a** and **b**: Percentages of CD3^+^CD4^+^IFN-γ^+^ (Th1) and CD3^+^CD8^+^IFN-γ^+^ (Tc1) gated from CD3^+^ cells analysed by FACS. **c**. IgG, IgG1 and IgG2a levels in mice sera were detected. Data are presented as the means ± SEM from eight mice in each group. (*P < 0.05; **P < 0.01)

In the conventional vaccination-challenge experiment, three immunisations with antigen are usually adopted. To assess the impact of Tat-coupled TPI vaccination on the immune system in mice, we analysed the percentages of CD4^+^IFN-γ^+^(Th1), CD8^+^IFN-γ^+^(Tc1) T cells in the spleens by flow cytometry. Results showed that the percentages of CD4^+^IFN-γ^+^ and CD8^+^IFN-γ^+^ T cells from IFA-immunised mice were similar to those in PBS controls, while both of them were increased significantly in Tat-TPI-immunised and TPI-immunised mice (*P* < 0.01). Moreover, we found that CD4^+^IFN-γ^+^ T-cell percentages were even higher in Tat-TPI group compared to TPI group (*P* < 0.05) (Fig. 4a, and b). These findings suggested that Tat-TPI immunisation could elicit a higher level of Th1 and Tc1 response at the same time.

Antibody responses including IgG, IgG1 and IgG2a antibody levels assayed by ELISA were also observed in addition to T-cell responses at two weeks after the third vaccination prior to challenge. High levels of IgG, IgG1 and IgG2a antibodies were detected in sera from mice vaccinated with Tat-TPI and TPI in comparison to IFA immunisation and PBS control (*P* < 0.01) (Fig. 4c). Both Tat-TPI and TPI immunisation elicited a strong IgG response in mice with little difference in IgG levels between them. However, there were apparent differences in IgG1 and IgG2a levels based on their OD values. Three immunisations with Tat-TPI produced more IgG1 than TPI immunisation (*P* < 0.05), while the opposite trend was seen in the IgG2a production (*P* < 0.01). The ratio of IgG2a/IgG1 in mice vaccinated with Tat-TPI and TPI were 0.63 and 1.59, respectively.

### Immune protection associated with Tat-TPI and TPI vaccination

**Fig. 5:**
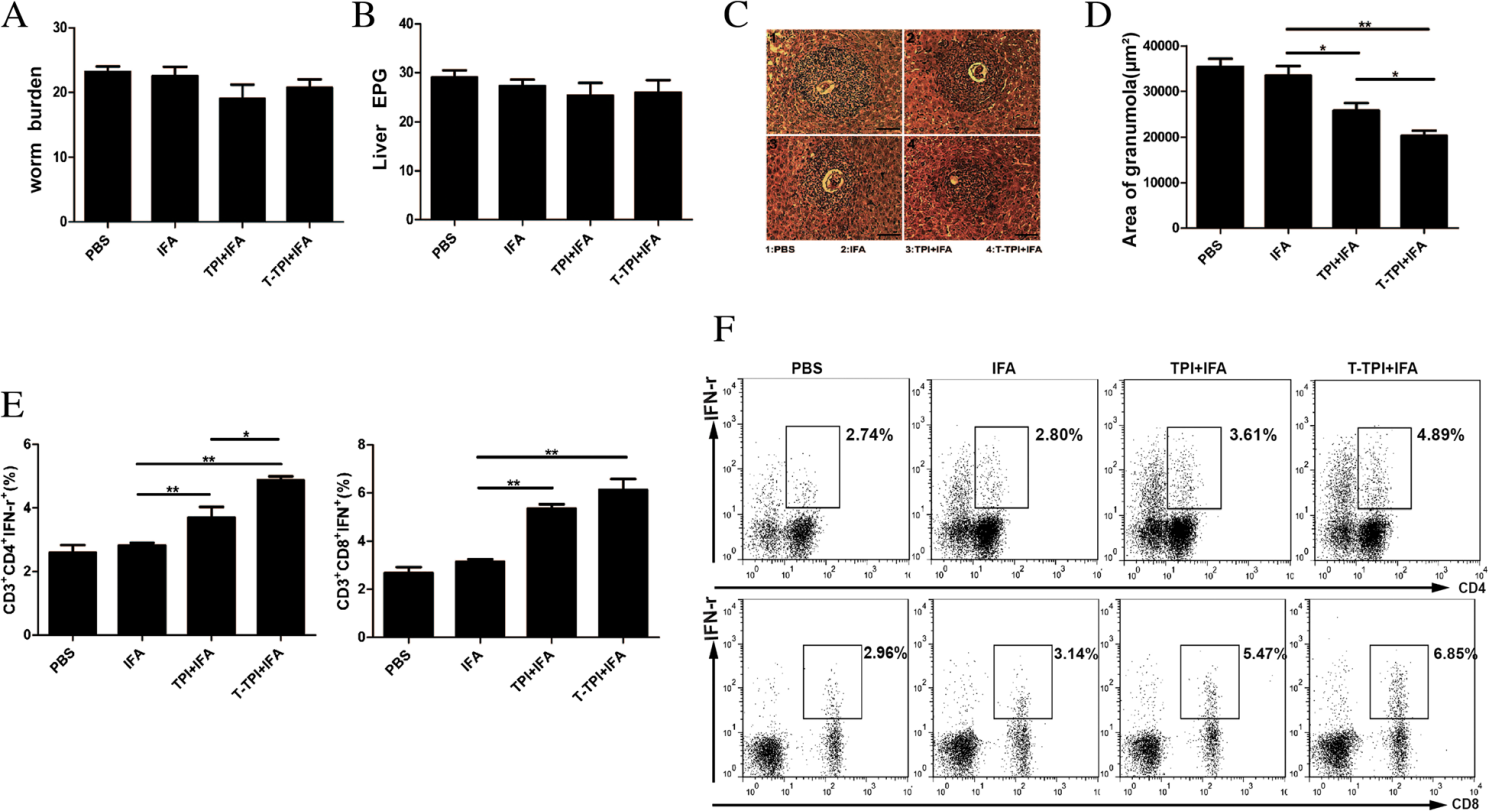
Parasite burden and immune response were observed at 6 weeks after *S. japonicum* infection in mice vaccinated with T-TPI + IFA, TPI + IFA, IFA and PBS. **a**. Average number of worms recovered. **b**. Average number of eggs per gram (EPG) in the liver. **c**. Representative granulomas with a single egg from each group (100×). **d**. Average area of single egg granulomas from each group. **e**, **f**. Percentages of CD3^+^CD4^+^IFN-γ^+^(Th1) and CD3^+^CD8^+^IFN-γ^+^(Tc1) gated from CD3^+^ T cells analysed by FACS. Each bar represents the means ± SEM from twelve mice per group. (*P < 0.05; **P < 0.01)

At six weeks after *S. japonicum* cercariae challenge, we calculated the numbers of adult worm recovered and eggs in the livers. Although the numbers of adult worms and eggs per gram (EPG) in the livers of mice vaccinated with TPI or Tat-TPI were slightly less than those in IFA immunisation and PBS control, there showed no significant differences among these four groups. Most importantly, these two antigens immunisation led to the smaller hepatic granuloma area with a single egg than IFA or PBS control groups with a significant difference. Especially, Tat-TPI-immunised mice showed the smallest granuloma area in the liver among the four groups (Fig. 5c, and d). The Th1 and Tc1 responses were detected by flow cytometry (Fig. 5e, and f). High levels of Th1 and Tc1 responses were detected in mice immunised with Tat-TPI and TPI in comparison to IFA immunisation and PBS control (*P* < 0.01). Results also showed that Th1 and Tc1 responses in mice immunised with TPI remained at high levels, which were still slightly lower than those of Tat-TPI-immunised mice, but there was no statistical significance in Tc1 reaction.

## Discussion

In this study, our initial conception was that the optimal cooperation among CD8^+^T cells, CD4^+^T cells and antibody responses would be required against schistosome infection. Thus, we adopted the protein transduction technology by using cell-permeable peptides to investigate the impact of different antigen-presented approaches on the host immune response and anti-infection protection.

The 11aa HIV-1 Tat was used to construct the fusion protein, Tat-TPI, in our study. HIV-Tat can activate CD8^+^T cells to produce high levels of IFN-γ. This concept is supported by some other results. Kronenberg et al. [19] reported that Tat-fusion proteins are superior in activating protective, CD8^+^T-cell response against *Leishmania major* when compared with antigen alone, and similar results were reported by Nicoli et al. [20] and Sforza et al. [21]. The increased CD8^+^T cells proliferation may be related to the improved expression of MHC-I complexes on the surface of DCs stimulated by Tat [22, 23]. Tat modulates the expression of T-box transcription factors T-bet [21] critical for stimulation of T-lymphocytes. In addition, it has been demonstrated that Tat could increase the secretion of IFN-α by macrophages [24] and IL-12 by DCs [25]. Our study showed that whether injected with Tat-TPI in foot pad or vaccinated with Tat-TPI in the back subcutaneously for three times, the draining lymph nodes and spleen both developed stronger CD8^+^T responses (Tc1) in the host.

It is the noteworthy finding that a higher Th1 level was observed in Tat-TPI vaccinated group in comparison to TPI immunisation. In order to better understand \whether the increased Th1 response was influenced by CD8^+^ T cells, we conducted anti-CD8a antibody-blocking assay in vitro. When CD8^+^T cells were blocked, Tat-TPI stimulated spleen cells expressed significantly lower levels of Th1 cells, indicating that CD8^+^T cells may assist CD4^+^T cells to produce more IFN-γ (Th1), which was in accordance with the study of Herath et al. [26]. CD8^+^T cells producing IFN-γ are important for regulating the CD4^+^T-cell response and Herath et al. [26] observed that the percentage of CD4^+^T cells producing IFN-γ was reduced with the depletion of CD8^+^T cells. In human leishmaniasis, da Silva Santos et al. [27] also found that CD8^+^T cells expressed activation markers at first and drove Th1 differentiation. On the other hand, CD8^+^T cells could also regulate IgG1 production [28]. We did realise that immunisation with Tat-TPI for three times was prone to produce IgG1 antibody subtype, while TPI vaccination was predominant to induce IgG2a production. In schistosome infection, IgG2a subclass is considered to be related to high protection [7, 29].

Generally, the studies of protective mechanisms against schistosomiasis are concentrated on CD4^+^T cells and antibody responses [6]. First, CD4^+^T-cell-produced IFN-γ is considered as the key factor [30, 31]. IFN-γ activates host macrophages to produce NO, which help eliminate parasites efficiently [10] . Secondly, the antibody response in attenuated vaccine-induced protection has been given much attention [9]. Yole et al. [32] reported that schistosome specific IgG antibody levels were positively correlated with the protection level against *S. mansoni* infection. However, the role of CD8^+^T cells played in schistosome infection, has been debated. In our study, we found that the increased CD8^+^T-cell response (Tc1) induced by Tat-TPI could not affect the development of adult worms, but helps to reduce significantly the hepatic granulomatous area with a single egg. Previous studies [33, 34] have shown that granulomatous reactions were gradually down-regulated due to the increase of CD8^+^T cells in the chronic phase of *Schistosoma mansoni* infection and the new granuloma formations were decreased significantly at 16–20 weeks after infection. When the spleen and lymph node cells of chronic phase were transferred to acute infection mice, newly formed granulomas in mice reduced significantly, and the inhibitory effect against granuloma response would disappear after depletion of CD8^+^T cells. Thus, in schistosome infection, CD8^+^T cells might regulate the immune pathological reaction *via* secreting some cytokines such as IFN-γ. Although high levels of Th1, Tc1 and IgG were induced by Tat-TPI immunisation, they did not achieve the ideal reduction rate of worm (11.9 %) and egg (12.4 %) burden in mice, being even lower than those in the TPI immunised group (21.9 % and 12.6 %, respectively). We have to admit that the protective mechanisms against schistosome infection are so complicated that we need to further explore the optimal vaccine strategy. Another possible cause is that mice model is not an ideal animal model to test vaccine efficacy, but is convenient to provide the relevant immunological observations.

In summary, this study suggests that immunisation with Tat-fused TPI may contribute to enhance CD4^+^ T-cell response and decrease hepatic egg granulomatous area after *S. japonicum* infection, but the anti-infection efficiency was limited. Thus, the optimal vaccine strategy is still the goal we are pursuing.

## Conclusions

Tat-coupled TPI immunisation could activate CD8^+^T cells in mice, which assist CD4^+^T cells to produce more IFN-γ. Also, it could boost IgG production, especially IgG1 subclass. Although this vaccine strategy with Tat-TPI immunisation did not turn out to be effective in decreasing parasite burden, it did reduce the single egg granuloma area in the livers after *Schistosoma japonicum* infection.
